# Supplementary material for: Discovery of New Secondary Metabolites from Marine Bacteria Hahella Based on an Omics Strategy
Source: Mar Drugs. 2022 Apr 18;20(4):269. doi: 10.3390/md20040269 (PMC9030710; doi:10.3390/md20040269)
Supplement: Supplementary file 1 [file marinedrugs-20-00269-s001.zip › marinedrugs-1677818-supplementary.pdf]

Supplementary Materials

Discovery of New Secondary Metabolites from Marine Bacteria *Hahella* Based on Omics-Strategy

Shufen He[#, 1], Peishan Li[#, 1], Jingxuan Wang[1], Yanzhu Zhang[1], Hongmei Lu[1], Liufei Shi[1], Tao Huang[2], Weiyan Zhang[1], Lijian Ding[1], Shan He[1,3], Liwei Liu[1,3*]

Author affiliations:

[1] Li Dak Sum Marine Biopharmaceutical Research Center, Department of Marine Pharmacy, College of Food and Pharmaceutical Sciences, Ningbo University, Ningbo, Zhejiang 315832, China;

[2] Department of Food Science and Engineering, College of Food and Pharmaceutical Sciences, Ningbo University, Ningbo 315211, P. R. China;

[3] Ningbo Institute of Marine Medicine, Peking University, Ningbo, Zhejiang 315800, China;

2011085032@nbu.edu.cn(S.H);196000818@nbu.edu.cn(P.L.);2111085014@nbu.edu.cn(J.W.);2111085060@nbu.edu.cn(H.L.);shiliufei@nbu.edu.cn(L.S.);2011085092@nbu.edu.cn(Y.Z.);huangtao@nbu.edu.cn(T.H.);zhangweiyan@nbu.edu.cn(W.Z.);dinglijian@nbu.edu.cn(L.D.); heshan@nbu.edu.cn(H.S.)

[*] Correspondence: Liwei Liu, liuliwei@nbu.edu.cn.

[#] S. He and P. Li contributed equally to this work.

Liwei Liu: ORCID 0000-0002-0578-1091

The Table of Contents

Table S1 The list of five *Hahella* genome 3

Table S2 The distribution of SMBGCs in six *Hahella* strains 3

Table S3 All prodiginine derivatives in *Hahella chejuensis* NBU794 4

Table S4 The standard curve of prodigision production 5

Table S5 The prodigiosin yield in *Hahella chejuensis* NBU794 grown in different mediums 6

Table S6 ^{1}H NMR and ^{13}C NMR data of chejuenolide A in CD_3OD 7

Figure S1 The structures of podigiosin, podiginine, chejuenolide A-C and lankacidin C 8

Figure S2 The SMBGCs of 'Other Types' identified in *Hahella* 9

Figure S3 The RiPPs identified in *Hahella* 10

Figure S4 Maximum likelihood phylogenetic tree of NBU794 and other strains in the genus *Hahella* ... 11

Figure S5 The SMBGCs predicated in *Hahella chejuensis* NBU794 12

Figure S6 Molecular networking of prodiginine derivatives in *Hahella chejuensis* NBU794 13

Figure S7 Prodigiosin in *Hahella chejuensis* NBU794 and *Hahella chejuensis* KCTC 2396 14

Figure S8. HPLC of the ethyl acetate extract of *H. chejuensis* NBU794 grown in M9 medium 15

Figure S9 ^{1}H and ^{13}C NMR of chejuenolide A recorded at 600 MHz in DMSO-d_6 16

Figure S10 HPLC analysis of chejuenolide A-C in *Hahella chejuensis* NBU794 and KCTC 2396 17

Figure S11 Two new prodiginine derivatives in this study and dipyrrolyldipyrromethene prodigiosin .. 18

Table S1. The list of five *Hahella* genome.

Strain name	Genome Size	GC%	INSDC	Sequence level
H. chejuensis KCTC 2396	7.22	53.9	CP000155.1	Complete
H. chejuensis. HN01	7.13	53.9	JAHMIN000000000.1	Contig
H. chejuensis. KA22	6.96	53.9	CP034836.1	Complete
H. ganghwensis DSM 17046	6.56	49.2	AQXX00000000.1	Contig
Hahella sp.CCB-MM4	6.66	49.8	MRYI00000000.1	Contig

Table S2. The distribution of SMBGCs in six *Hahella* strains. *Hahella ganghwensis* DSM 17046, *Hahella chejuensis* KCTC 2396, *Hahella chejuensis*. KA22, *Hahella chejuensis*. HN01, *Hahella* sp.CCB-MM4, and *Hahella chejuensis* NBU794.

Strain / Gene cluster	***H. chejuensis* HN01**	***H.chejuensis*. KA22**	***H. ganghwensis* DSM 17046**	***Hahella* sp. CCB-MM4**	***H. chejuensis* KCTC 2396**	**Total**
**betalactone**	1	1	1	1	1	5
**NRPS**	4	5	1	1	4	15
**NRPS/PKS**	3	4	1	5	4	17
**NAGGN**	1	1	1	1	1	5
**ectoine**	1	1	-	1	1	4
**thioamide-NRP**	1	1	-	-	1	3
**CDPS**	-	-	1	-	-	1
**hserlactone**	-	-	1	1	-	2
**siderophore**	-	-	1	1	-	2
**butyrolactone**	-	-	1	-	-	1
**PBDE**	-	-	-	1	-	1
**RiPPs**	4	4	1	1	4	14
**Total**	15	17	9	13	16	70

Table S3. All prodiginine derivatives in *Hahella chejuensis* NBU794.

**Peak #**	**Molecular ion (m/z, [M+H]$^+$)**	**Compound formula**	**λ (nm)**	**Identification**
1	296.1759	$C_{18}H_{21}N_3O$	534	2-methyl-3-propyl-prodiginine
2	340.2015	$C_{20}H_{25}N_3O_2$	530	2-methyl-3-propyl-4-O-methyl-prodiginine
3	354.2171	$C_{21}H_{27}N_3O_2$	528	2-methyl-3-pentyl-4-O-methyl-prodiginine
4	338.2216	$C_{21}H_{27}N_3O$	534	2-methyl-3-hexyl-prodiginine
5	366.2178	$C_{23}H_{31}N_3O$	532	2-methyl-3-octyl-prodiginine
6	394.1896	$C_{25}H_{35}N_3O$	528	Undecylprodiginine
7	324.2067	$C_{20}H_{25}N_3O$	534	prodigiosin
8	310.1909	$C_{19}H_{23}N_3O$	528	2-methyl-3-butyl-prodiginine
9	352.2380	$C_{22}H_{29}N_3O$	534	2-methyl-3-heptyl-prodiginine

Table S4: The standard curve of prodigision production. The different concentrations of prodigision solutions are made by proper dilution and injected into Agilent 1260 HPLC for quantification. The UV peak areas at UV 530nm are integrated with Agilent ChemStation software. The prodigision standard curve is made by introducing concentration of prodigision as ‘x’ and peak area as ‘y’ to form the calculation formular.

Concentration (mg/mL)	Peak area (λmax=530nm)
0.0390625	5353.8
0.078125	10224
0.15625	20939.7
0.3125	34381.9
0.625	68439.8
1.25	97278.2
2.5	154444

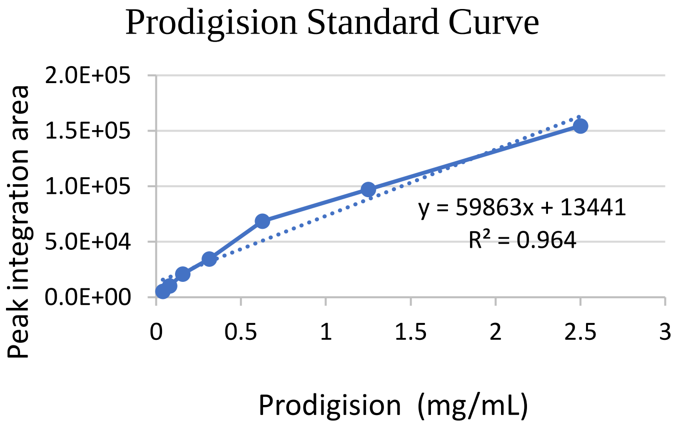

Table S5. The prodigiosin yield in *Hahella chejuensis* NBU794 grown in different mediums. ISP4: standard ISP4 medium; ISP4+HP20: standard ISP4 medium added with HP20 beads; ISP4+HP20+Glucose: modified ISP4 medium (starch is replaced with equal amount of glucose) added with HP20 beads; ISP4+HP20+Sucrose: modified ISP4 medium (starch is replaced with equal amount of sucrose) added with HP20 beads. The peak areas are quantified through HPLC analysis at UV 530nm. The yields are calculated based on the peak integration area and prodigision standard curve (**Table S5**).

Name	Peak integration area	Prodigiosin yield (mg/mL)
ISP4	18023.5	1.40
ISP4+HP20	218805	3.43
ISP4+HP20+Glucose	220400.7	3.46
ISP4+HP20+Sucrose	362447.3	5.83

Table S6. ^{1}H NMR and ^{13}C NMR data of chejuenolide A in CD_3OD

Position	chejuenolide A		chejuenolide A (in this study)	
	δ_C	δ_H (*J* in Hz)	δ_C	δ_H (*J* in Hz)
**1**	205.6		205.6	
**2**	44.9	3.40（1H,dq,10.3,6.6）	44.9	3.40(1H, dq,10.3,6.6)
**3**	51.0		51.0	
**4**	131.6	5.08(1H, d,10.3)	131.6	5.08(1H, d,10.3)
**5**	135.5		135.5	
**6**	136.7	5.74 (1H, d, 15.9),	136.8	5.74 (1H, d, 15.9)
**7**	131.3	5.36 (1H, dd,15.9,8.0)	131.3	5.36 (1H, dd,15.9,8.0)
**8**	74.8	4.04(1H, ddd, *10.6*, 8.0,4.0)	74.8	4.04(1H, ddd, *10.6*, 8.0,4.0)
**9a**	36.2	2.45 (1H, m)	36.7	2.49 (1H, m)
**9b**		2.17 (1H, ddd, *12.9*, 10.6,8.7)		2.17 (1H, ddd, *12.9*, 10.6,8.7)
**10**	127.7	5.18(1H, t,8.7)	127.7	5.18(1H, t,8.7)
**11**	135.7		135.7	
**12**	135.3	6.04(1H, d,15.7)	135.3	6.04(1H, d,15.7)
**13**	129.6	5.42(1H, dd,15.7,5.2)	129.6	5.42(1H, dd,15.7,5.2)
**14**	71.3	4.52 (1H, m)	71.3	4.52 (1H, m)
**15a**	37.4	2.72(1H, ddd,14.0,10.3,3.2)	37.4	2.72(1H, ddd,14.0,10.3,3.2)
**15b**		2.55(1H, m)		2.51(1H, m)
**16**	139.2	6.70 (1H, dd, *10.3,5.3*)	139.2	6.70 (1H, dd, *10.3,5.3*)
**17**	140.0		140.0	
**18**	16.1	0.99 (3H, d, *6.6*)	16.1	0.99 (3H, d, *6.6*)
**19**	12.8	1.68(3H, d,1.1)	12.8	1.68(3H, d,1.1)
**20**	13.6	1.58(3H, s)	13.6	1.58(3H, s)
**21**	12.5	1.71(3H, br s)	12.5	1.72(3H, br s)
**1'**	172.4		172.4	
**2'**	22.7	1.93(3H, s)	22.7	1.93(3H, s)

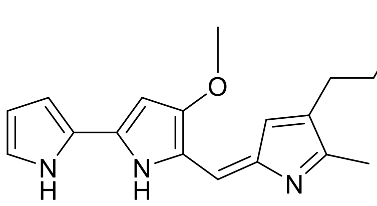

Prodigiosin

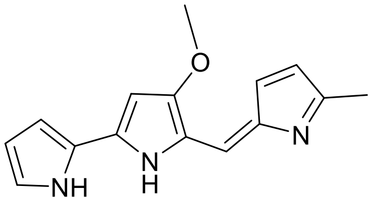

Prodiginine

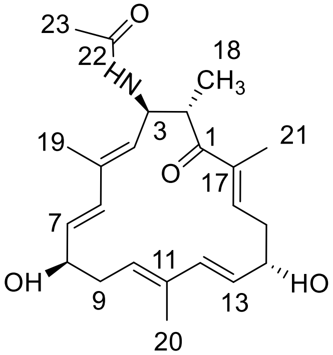

Chejuenolide A

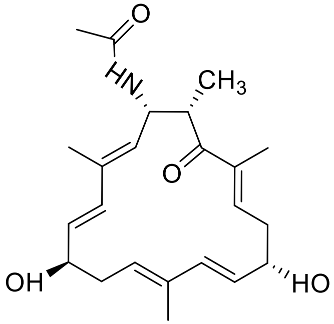

Chejuenolide B

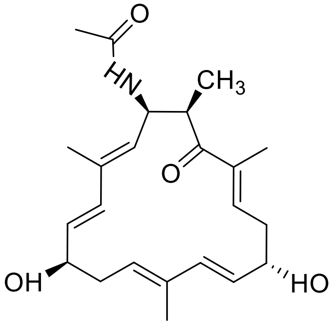

Chejuenolide C

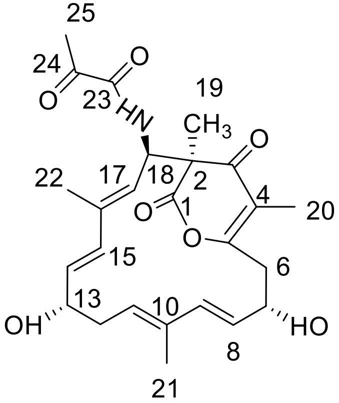

Lankacidin C

Figure S1. The structures of podigiosin, podiginine, chejuenolide A-C, and lankacidin C.

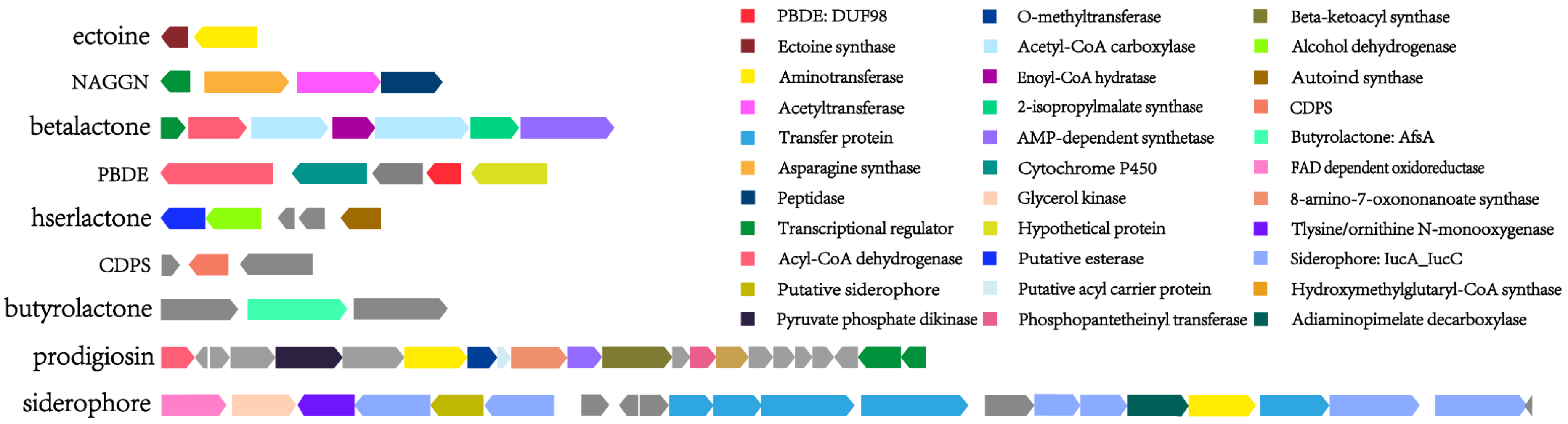

Figure S2. The SMBGC of 'Other Types' identified in *Hahella*. The functional genes are indicated by squares with different colors.

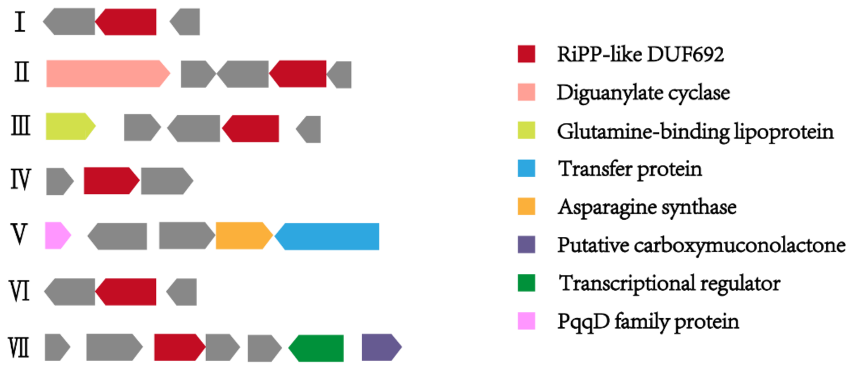

Figure S3. The RiPPs (ribosomally synthesized and post-translationally modified peptide biosynthetic gene clusters) identified in *Hahella*. All gene clusters were classified into seven groups based on the gene composition. The functional genes were indicated by squares with different colors.

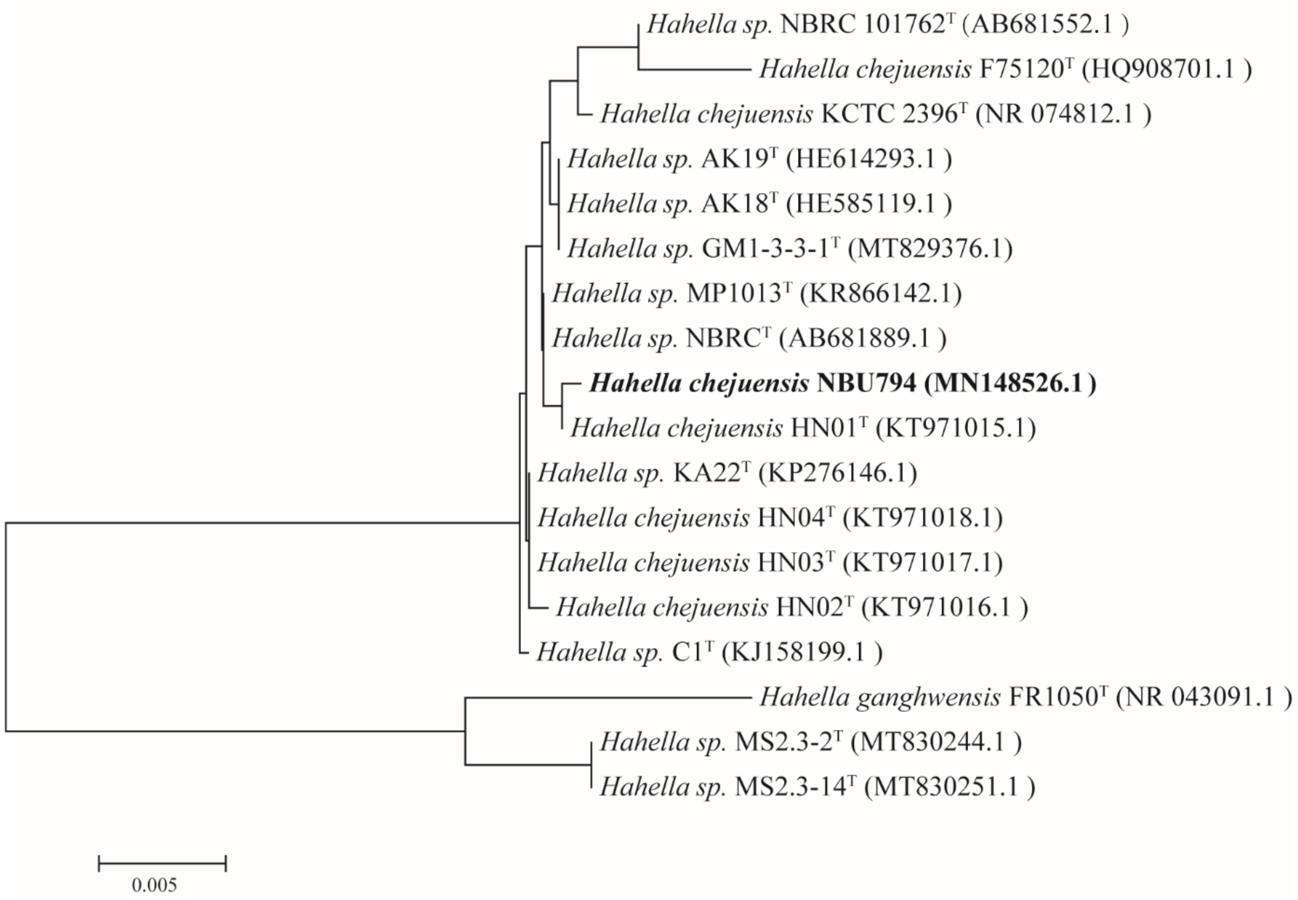

Figure S4. Maximum likelihood phylogenetic tree of NBU794 and strains in the genus *Hahella* based on 16S rRNA gene. Bar, 0.005 represents nucleotide substitution rate (Knuc) units. Strains *H. chejuensis* NBU794 from this study are highlighted in bold.

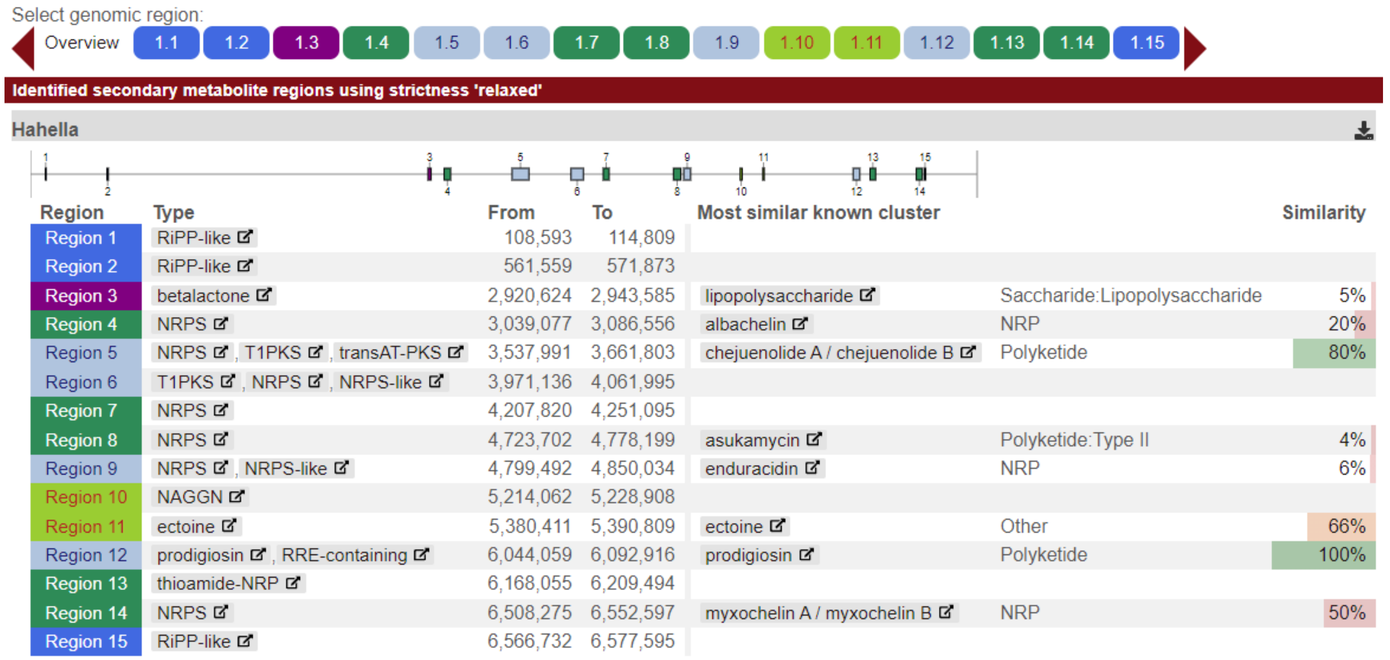

Region	Type	From	To	Most similar known cluster		Similarity
Region 1	RiPP-like	108,593	114,809			
Region 2	RiPP-like	561,559	571,873			
Region 3	betalactone	2,920,624	2,943,585	lipopolysaccharide	Saccharide:Lipopolysaccharide	5%
Region 4	NRPS	3,039,077	3,086,556	albachelin	NRP	20%
Region 5	NRPS, T1PKS, transAT-PKS	3,537,991	3,661,803	chejuenolide A / chejuenolide B	Polyketide	80%
Region 6	T1PKS, NRPS, NRPS-like	3,971,136	4,061,995			
Region 7	NRPS	4,207,820	4,251,095			
Region 8	NRPS	4,723,702	4,778,199	asukamycin	Polyketide:Type II	4%
Region 9	NRPS, NRPS-like	4,799,492	4,850,034	enduracidin	NRP	6%
Region 10	NAGGN	5,214,062	5,228,908			
Region 11	ectoine	5,380,411	5,390,809	ectoine	Other	66%
Region 12	prodigiosin, RRE-containing	6,044,059	6,092,916	prodigiosin	Polyketide	100%
Region 13	thioamide-NRP	6,168,055	6,209,494			
Region 14	NRPS	6,508,275	6,552,597	myxochelin A / myxochelin B	NRP	50%
Region 15	RiPP-like	6,566,732	6,577,595			

Figure S5. The SMBGCs predicated in *Hahella chejuensis* NBU794 with anti-SMASH 6.0.

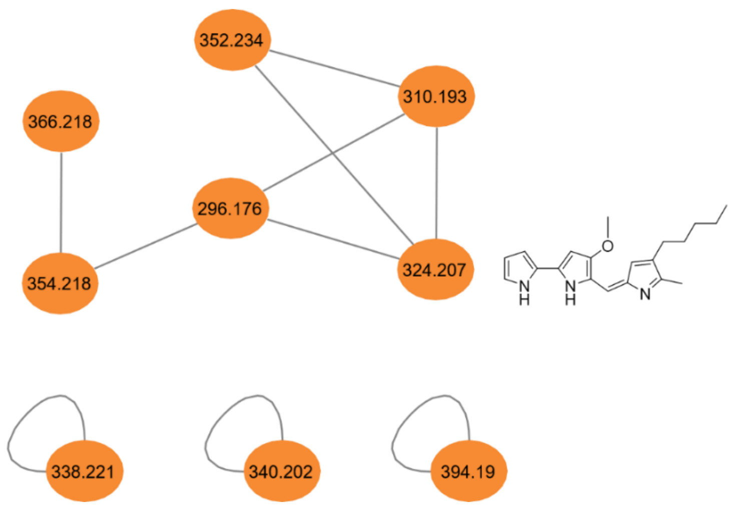

Figure S6. Molecular networking of prodiginine derivatives in *Hahella chejuensis* NBU794. Each node indicates one compound with molecular ion inside. The node marked with 324.207 is prodigiosin, and its structure is listed on the right.

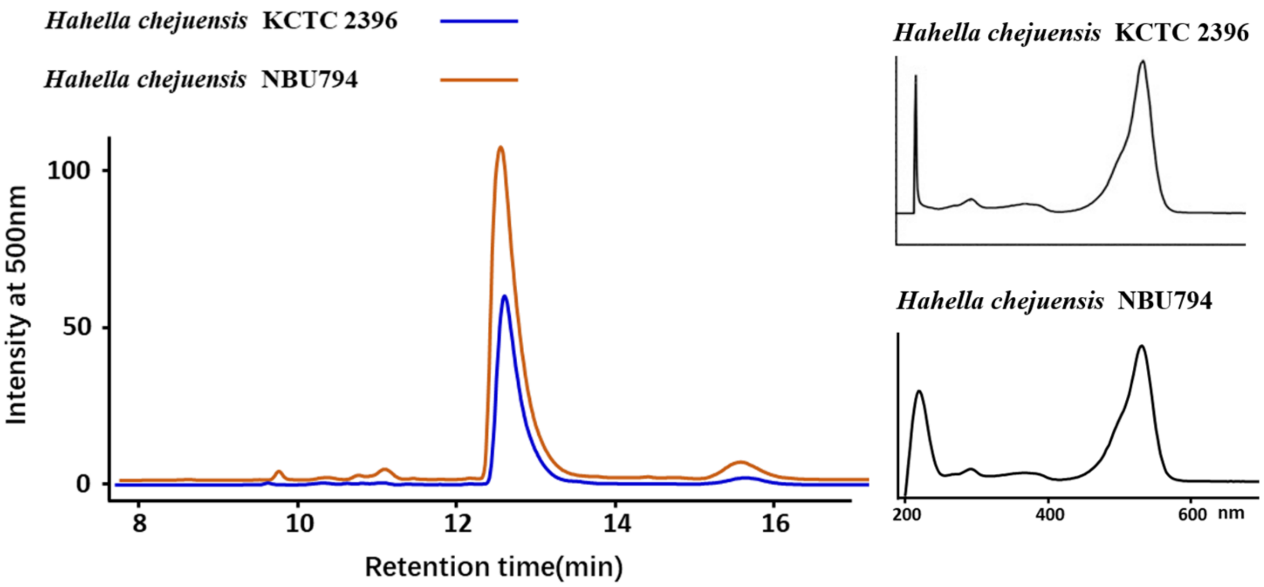

Figure S7. HPLC analysis of prodigiosin *in Hahella chejuensis* NBU794 and *Hahella chejuensis* KCTC 2396.

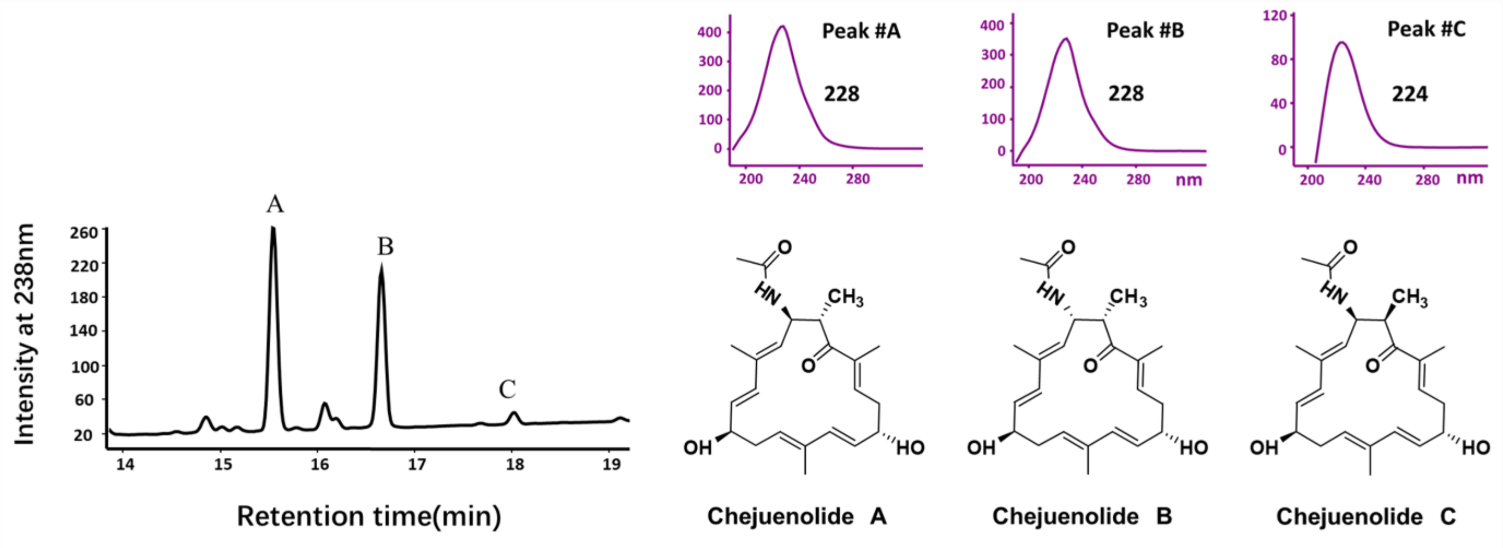

Figure S8. HPLC chromatogram of the ethyl acetate extract of *H. chejuensis* NBU794 grown in M9 medium. The UV−Vis spectra of chejuenolide A-C and their structures are listed on the right.

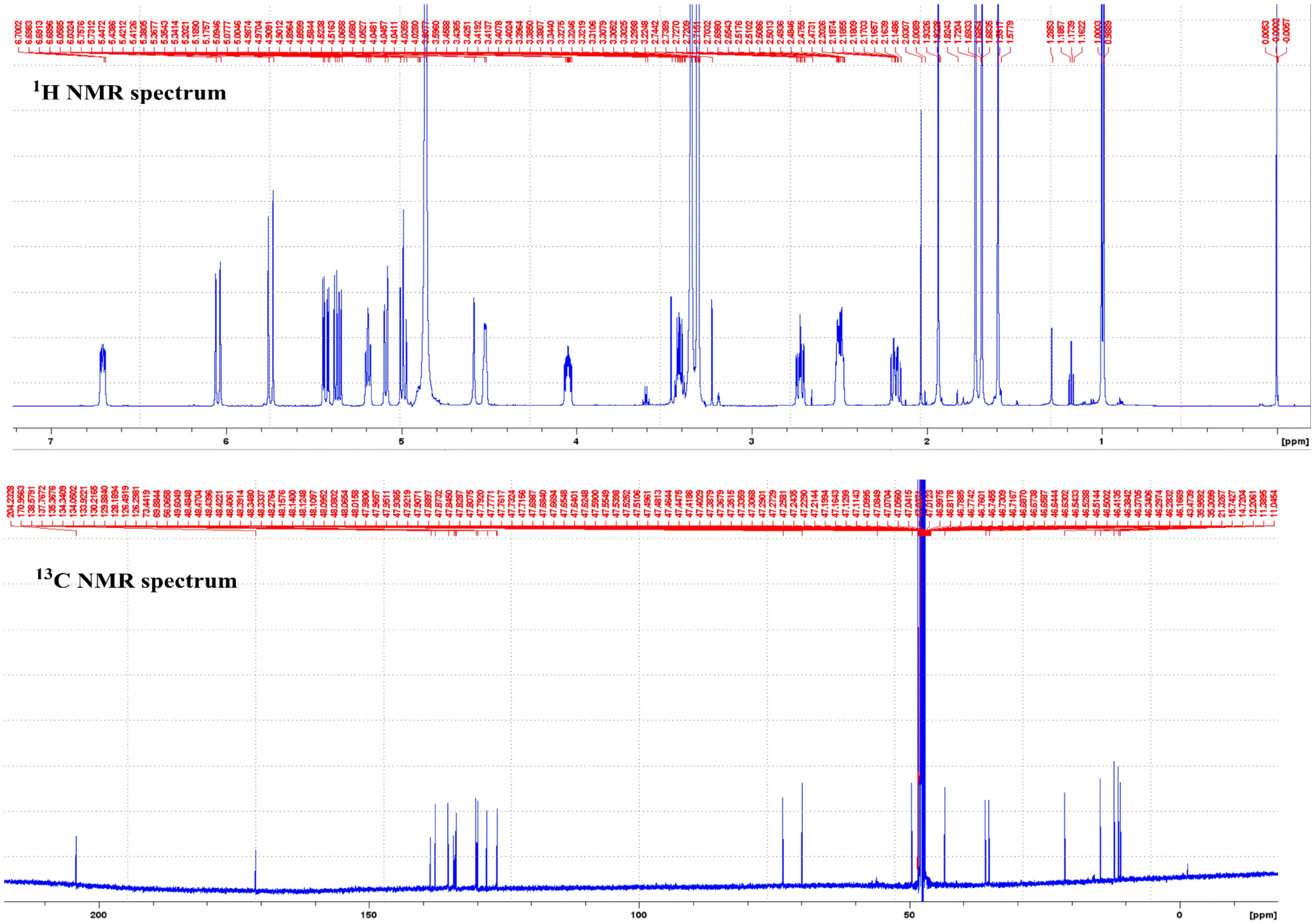

Figure S9. ^{1}H and ^{13}C NMR of chejuenolide A recorded at 600 MHz in DMSO-d_6.

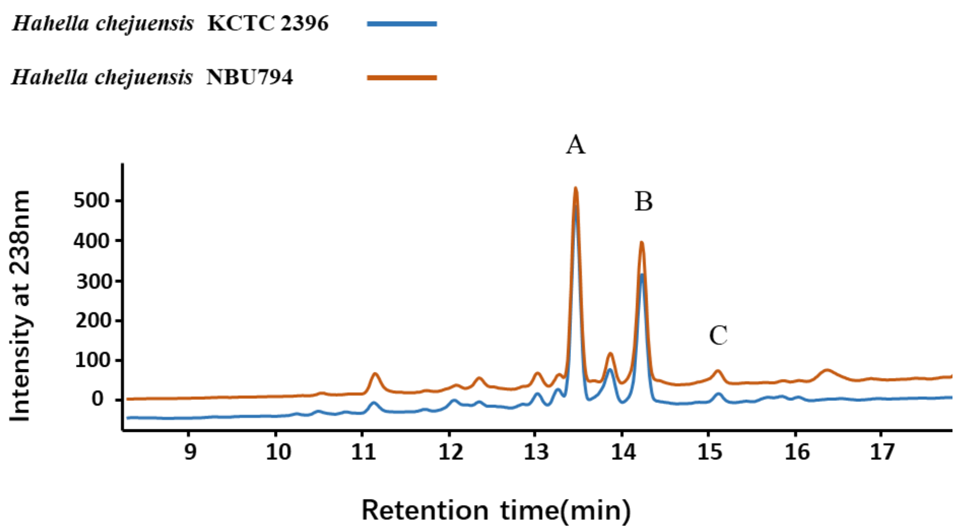

Figure S10. HPLC analysis of chejuenolide A-C *in Hahella chejuensis* NBU794 and *Hahella chejuensis* KCTC 2396.

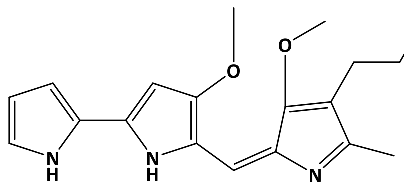

2-methyl-3-propyl-4-O-methyl-prodiginine

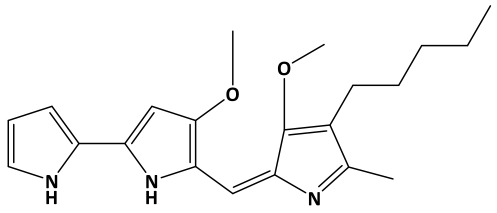

2-methyl-3-pentyl-4-O-methyl-prodiginine

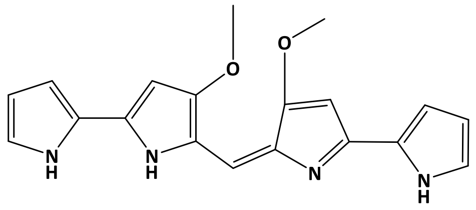

Dipyrrolyldipyrromethene prodigiosin

Figure S11. Two new prodiginine derivatives in this study and dipyrrolyldipyrromethene prodigiosin.
